# Early outcomes of transcatheter arterial embolization using imipenem/cilastatin for plantar fasciitis refractory to conservative therapy

**DOI:** 10.1093/bjr/tqae012

**Published:** 2024-01-27

**Authors:** Rozil Gandhi, Mohal Banker

**Affiliations:** Health1 Hospital, Ahmedabad, Gujarat 380059, India; Sushrut Hospital, Ahmedabad, Gujarat 380007, India; Health1 Hospital, Ahmedabad, Gujarat 380059, India; Bankers Vascular Hospital, Ahmedabad, Gujarat 380015, India

**Keywords:** plantar fasciitis, embolization, TAE, VAS score

## Abstract

**Objective:**

The conservative therapy for chronic plantar fasciitis works for a few patients, while surgical options have drawbacks. Before considering surgical options, transcatheter arterial embolization may help patients with plantar fasciitis who are experiencing discomfort resistant to conservative treatment.

**Methods:**

We report evaluation data of 10 patients treated with transcatheter arterial embolization using imipenem/cilastatin as embolic agents to relieve chronic pain due to plantar fasciitis. All the patients were refractory to conservative therapy.

**Results:**

The technical success of the procedure was found to be 100%. Further, effective pain relief was observed as there was no pain relapse in 6 months, and patients did not require any other form of therapy.

**Conclusion:**

This report warrants further adequately designed randomized clinical studies for evaluating the efficacy of transcatheter arterial embolization in plantar fasciitis.

**Advances in knowledge:**

Resorting to surgical option for chronic pain relief in plantar fasciitis might be reconsidered and replaced with arterial embolization. However, adequately designed long-term clinical studies are required to prove its long-term efficacy.

## Introduction

Plantar fasciitis, the most common cause of heel pain, is a degenerative musculoskeletal condition involving the fascia that runs from the metatarsophalangeal joint to the calcaneal bone. It is expressed as intense heel pain, especially for the initial few steps in the morning. However, the pain subsides as normal physical activities are resumed.^1^ Plantar heel pain is commonly observed in physically active and sedentary individuals^2^ and more widely in females.^2^^,^^3^ A study in India found that 59% of the patients reported plantar heel pain due to calcaneal outgrowth.^3^

The pathology and aetiology of plantar fasciitis are multifactorial and not yet completely understood.^2^ Numerous conservative treatment options are prescribed for plantar fasciitis that involve rest, physical therapy, stretching exercises, orthosis (heel pads, heel wedges, heel cups, splints, magnetic insoles), ultrasound, non-steroidal anti-inflammatory drugs (NSAIDs), local steroid injections, platelet-rich plasma injections, extracorporeal shockwave therapy, or radiofrequency electromagnetic field therapy.^1^ Over 90% of individuals with plantar fasciitis experience symptomatic alleviation after 3-6 months of conservative treatment, making it a self-limiting illness. However, a small proportion of patients with chronic, severe, persistent symptoms for at least 6-12 months, refractory to conservative treatment, are advised surgery.^1^ Further, it is imperative to note that different techniques of surgeries have success only between 75% and 90%.^4^

Pain in chronic plantar fasciitis is linked to increased fascial vascularity.^5^ Transcatheter arterial embolization (TAE) involves selective blocking of blood supply to an abnormal tissue area. It has been considered an alternative to surgery for many clinical conditions, especially liver cancer.^6^ Recently, TAE has also been reported to be effective in musculoskeletal disorders such as osteoarthritis, tendinopathy, and enthesopathy, including plantar fasciitis.^7^^,^^8^ The basic idea is that the diseased tissue will complicate if the vascular supply is cut off. As a result, this tissue will no longer stimulate nociceptive responses or release pro-inflammatory mediators, and pain will subside. Thus, TAE may have a role in alleviating pain in patients who are refractory to conservative treatment for plantar fasciitis before surgical options are considered. Further, since imipenem/cilastatin is only marginally soluble in water, they have embolic effects when suspended in contrast media. Also, imipenem/cilastatin has a better safety profile than traditional agents such as gelatin.^9^

Therefore, we postulated that TAE using imipenem/cilastatin might relieve pain associated with chronic plantar fasciitis. Accordingly, the procedure was employed in some of our patients. The outcomes of these patients provided with such therapy at our centre are provided in the current report.

## Methods

### Patients

The present report encompasses the outcomes of the patients who have undergone the TAE procedure registered with the hospital’s committee in line with local policy. The presented data were recorded as part of a service evaluation and audit. Thus, formal ethics approval was not procured. All the data presented were made anonymous. Patients with chronic plantar fasciitis were given TAE therapy at our centre. Plantar fasciitis was confirmed in the patients by clinical examination, ultrasonography, and X-ray imaging. From June 2021 to July 2022, 10 patients with a total of 13 TAE procedures were included in this evaluation. All the patients selected for the TAE procedure were refractory to other conservative treatments for 3 months, such as anti-inflammatory drugs, stretching and strengthening exercises, corticosteroid injections, and physical therapy.

The patients were explained all the potential benefits and outcomes, risks, and side effects involved with the TAE procedure and the off-label use of imipenem/cilastatin. Written informed consent was obtained from all the patients. The symptomatic leg, as described by the patient, was chosen for the therapy. Exclusion criteria included local infection, coagulopathy, or any recent trauma. A total of 10 patients’ data is included in the evaluation (Table 1).

**Table 1.** Clinical data and procedural details of the patients.

Foot no.	Age	Sex	Pain duration (months)	Puncture site	Targeted arteries	Embolic agent volume (mL)
1	44	F	12	L CFA	L PTA	3
2	60	F	6	L CFA	L PTA	2
3	45	F	36	R CFA	R PTA	2.5
4	45	F	36	L CFA	L PTA	2.5
5	36	F	12	R CFA	R PTA	3
6	36	F	12	L CFA	L PTA	3
7	58	F	9	R CFA	R PTA	3
8	56	F	12	R CFA	R PTA	3
9	41	F	6	R CFA	R PTA	2
10	65	F	6	L CFA	L PTA	2
11	50	F	6	L CFA	L PTA	2
12	60	F	24	R CFA	R PTA	3
13	60	F	24	L CFA	L PTA	3

Abbreviations: R = right, L = left, CFA = common femoral artery, PTA = posterior tibial artery.

### Embolization procedure

The TAE was performed by the expert from our centre on all the patients. Using the Seldinger technique, ultrasound-guided 4-F paediatric sheath (Merit Medical Inc., United States) insertion in the common femoral artery of the affected limb was done anterogradely. Local anaesthesia (10 mL, 2% lignocaine, Loxicard^®^, Neon Laboratories, India, with 24G 1.5-in hypodermic needle) was injected prior. All the patients were administered 3000 IU heparin sodium for a unilateral procedure and 5000 IU heparin sodium (Zorparine^®^, 5000 IU/5 mL, Zorg Lifesciences, India) for a bilateral procedure from the vascular sheath. 4-F JR catheter (Cordis) was placed in the popliteal artery, and the posterior tibial artery was cannulated for the procedure in all the patients with 2-F progreat microcatheter introduced coaxially from 4-F catheter (Table 2). If the artery showing hypervascular blush could be cannulated, then super selective cannulation was done in that artery. However, if the artery could not be cannulated, the posterior tibial artery just prior to the artery showing blush was cannulated. After that, 3-5 mL of iodinated contrast media in 50% dilution (Ultravist^®^, iopromide 370 mgI/ml, Bayer Healthcare Pharmaceuticals, Germany) were manually injected to perform digital subtraction angiography. A microcatheter (Terumo, Tokyo, Japan) was explicitly implanted in the posterior tibial artery after the aberrant neovessels at the calcaneal attachment of plantar fascia were discovered (Table 2). Once the blood flow had stopped, 0.5 mL increments of an imipenem/cilastatin suspension (Imicrit^®^, 500 mg, Cipla, Mumbai, India) in iodinated contrast agent (7 mL) were administered, prepared by pumping syringes for 10 s. The injection was stopped once the hypervascular blush disappeared. Hence, the parent artery remains patent with just embolization of abnormal blush. Manual compression of the femoral puncture site for 20 min was used to achieve haemostasis, followed by 6 h of bed rest. The patients were discharged and walked home on the same day.

**Table 2.** VAS score measured at baseline and follow-up.

Foot no.	Previous Steroid therapy	Follow- up duration (months)	Pain score (VAS)^a^				
			Baseline	1-month Follow-up	3-month Follow-up	6-month Follow-up	12-month follow up
1	No	13	9	2	1	1	1
2	No	12	9	1	1	1	1^c^
3	No	9	8	1	1	1	1^c^
4	No	9	8	1	1	1	1^c^
5	Yes	8	8	6	2	1	1^c^
6	Yes	8	8	6	2	1	1^c^
7	Yes	7	9	2	1	1	1^c^
8	No	7	9	1	1	1	1^c^
9	No	3	8	0	0	1^c^	1^c^
10	Yes	3	9	1	1	1^c^	1^c^
11	Yes	3	9	2	2	1^c^	1^c^
12	No	3	8	1	1	1^c^	1^c^
13	No	3	8	1	1	1^c^	1^c^
Total			8.5 ± 0.52	1.9 ± 1.89^b^	1.2 ± 0.55^b^	1.00 ± 0.0^b^	1.00 ± 0.0^b^
*P*-value				.001	.001	.001	.001

a Using visual analog scale (VAS) 0 to 10.

b Statistically significant difference between baseline and follow-up.

c The VAS score was obtained via a telephonic follow-up. The emoji-based VAS scale was shared electronically with the patient, and VAS score assessment was conducted.

### Post-procedural care

All the patients could resume their normal physical activity from the next day of the procedure. They were given non-steroidal anti-inflammatory agents for 5 days after the procedure. However, they were advised not to perform any heavy lifting or unusual strenuous activities for up to 1 month. Further, stretching exercises at home after the procedure were also not recommended.

### Evaluation

The effectiveness of the TAE procedure was evaluated by measuring the changes in the visual analogue scale (VAS) score. The VAS score was measured before the treatment and after 1, 3, 6, and 12 months of the procedure on an outpatient basis. The degree of pain was recorded on a VAS score of 0-10, with 0 as no pain and the worst patient’s pain experience as 10.^10^ The VAS score was recorded as subjective pain experience using an emoji-based pain scale. This scale is composed of six icons that correspond to a numeric rating scale of 0-10. The patient points to the emoji that best depicts their pain experience.^11^ However, since all the patients had not completed the 12-month follow-up period, the data presented here include the VAS scores measured to 1 and 3 months for 10 patients, 6 months for 6 patients, and 12 months for 1 patient. The patients for whom the 12-month follow-up was not completed were contacted via a telephone call to assess their VAS score. The emoji-based pain scale used for the VAS scoring was shared electronically to assess the pain scoring. In addition, digital subtraction angiography was performed to observe the abnormal vessels before and after the treatment. Technical success was defined based on quality improvement guidelines for percutaneous transcatheter embolization by the Society of Interventional Radiology.^12^ It includes a variable degree of blood flow cessation or decreases to the lesion or target organ due to embolization, measured via complete angiography immediately after the procedure.^9^^,^^12^

### Statistical analysis

Data were recorded prospectively at baseline and on an ongoing basis at the clinic follow-up visits. The data were collated into an Excel spreadsheet (MS Excel, MS Office 365, Microsoft, Redmond, WA, United States) and analysed using SPSS (version 25.0). The baseline data and data from the follow-up appointments were compared. The analysis was performed using non-parametric tests, typically the Wilcoxon signed-rank test, to study the pre- and post-treatment differences. Statistical significance was set at *P* < .05.

## Results

The TAE therapy was provided to 10 patients. Three of the patients were provided with therapy in both legs. Thus, a total of 13 TAE procedures were conducted. The mean age of the patients was 51.5 ± 9.66 years (mean ± standard deviation [SD]), and the mean duration of symptoms was 12.9 ± 9.80 months (mean±SD). The most prolonged duration of symptoms reported was 36 months, while the shortest was 6 months. The clinical data of the patients are presented in Table 1.

Five of the 10 patients had received steroid injections before initiating the therapy (Table 2). Before applying TAE therapy, a minimum period of 3 months was kept after administering the last steroid injection. The other patients were not found suitable for the steroid injections since it may cause weakness in the tendons. Thus, the patients were provided with TAE therapy after the other conservative therapies failed. None of the patients required any other treatment after the TAE procedure during their follow-up. Furthermore, the patients also reported pain resolution when followed telephonically for 12-month follow-up (Table 2). Besides, no significant side effects or complications were observed in any patient.

**Figure 1.**
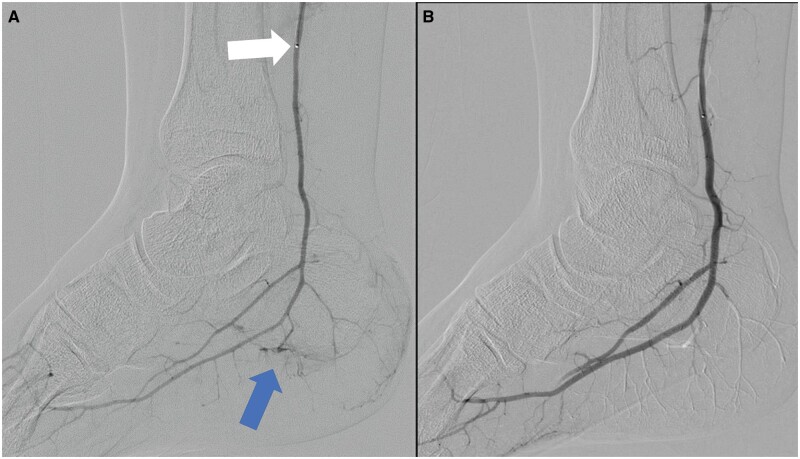
(A) Angiogram of distal right posterior tibial artery with microcatheter (white arrow) showing hypervascular abnormal blush (blue arrow) from a tiny branch supplying the insertion of plantar fascia. (B) No hypervascular blush noted after embolization on digital subtraction angiogram.

**Figure 2.**
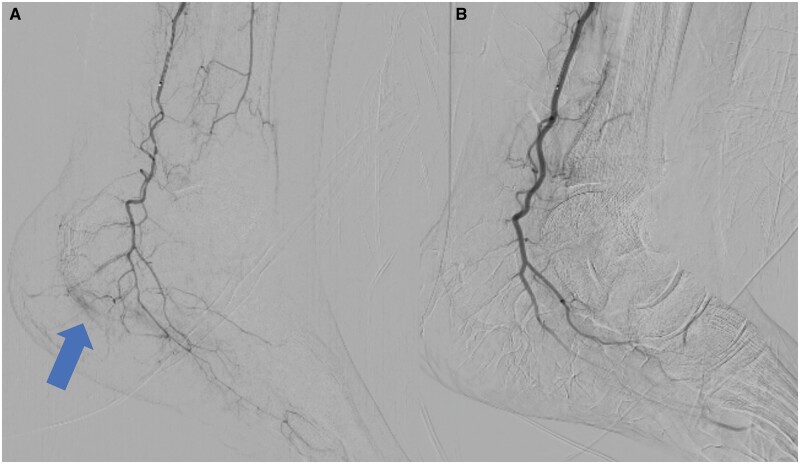
(A) Angiogram of distal left posterior tibial artery with microcatheter showing hypervascular abnormal blush (blue arrow) from a tiny branch supplying the insertion of plantar fascia at calcaneum. (B) No hypervascular blush noted after embolization on digital subtraction angiogram.

Technical success was observed in 100% of patients, including all 13 procedures conducted. Figures 1 and 2 depict the digital subtraction angiography images before and after the embolization procedure. We injected imipenem/cilastatin via a microcatheter. The VAS scores were significantly decreased from 8.5 ± 0.52 at baseline to 1.0 ± 0.00 (*P* = .001) at 12 months follow-up (Table 2).

## Discussion

The present report highlights the effectiveness of TAE in patients with chronic plantar fasciitis refractory to other conservative management. Treatment of such patients is challenging even though the disease is considered a self-limiting condition. Corticosteroid injections offer some benefits in relieving pain; however, it is usually short term. Furthermore, no quality evidence exists for the long-term benefit of corticosteroid injections. Nevertheless, a systematic review has also suggested corticosteroids to be equally efficacious to placebo injections.^13^

Furthermore, another meta-analysis found that other non-invasive treatment options like shockwave therapy or radiation therapy also provide similar short-term pain relief as corticosteroids.^14^ Besides, surgical options involve an increased risk of complications and long recovery periods.^15^ Thus, more effective, alternative, and long-term non-invasive techniques are required in the current situation to treat this disabling condition.

Transcatheter arterial embolization, a novel technique, has the potential to revolutionize clinical care and change how certain musculoskeletal disorders such as osteoarthritis, tendinopathy, myalgia, shoulder and neck pain, or plantar fasciitis are currently treated. Studies have reported its efficacy in controlling pain for more than 24 months in several musculoskeletal disorders.^9^ The exact mechanism of pain relief due to TAE with imipenem/cilastatin is still not known; however, blood flow reduction at the site of pathology may be one of the reasons for the same.^8^ Being a minimally invasive technique with long-term benefits, TAE potentially presents an alternative treatment for plantar fasciitis patients with pain refractory to other conservative and traditional therapies.

Some instances of the effectiveness of TAE have been reported for treating plantar fasciitis. Okuno et al reported a successful case of persistent plantar fasciitis treated by TAE that was refractory to NSAIDs and corticosteroid injections.^8^ Furthermore, Shibuya et al also reported a case report of a patient with plantar fasciitis treated with TAE. The pain for the patient decreased significantly, and she was able to perform all daily activities by the end of 3 months.^16^ The present study reports 13 successful cases of TAE with chronic pain due to plantar fasciitis. All the patients in our study were satisfied with the pain relief and had been free from painful feelings for a minimum period of 6 months. The angiographic findings of our patients suggested successful devascularization at the calcaneal joints (Figures 1 and 2). This may be responsible for the reduction in pain. Moreover, none of our patients reported any complications such as ischaemia, ulcers, necrosis, or fascia rupture.

The current evaluation does not constitute a properly designed clinical study and presents the cases evaluated as service audits. Thus, the demographic or risk factors associated with the patients may vary and pose bias. Furthermore, the patient cohort was petite with a short follow-up period. The follow-up of 12 months was not completed in all patients. Most of our patients were from remote geographies and visiting tier 1 cities for advanced management. The follow-ups were difficult in this Indian setting considering out-of-pocket expenses for travel and follow-up consultations. Hence, continuous follow-ups were difficult and a limitation in our study. However, we managed to conduct the long-term follow-ups via a telephone call. Thus, we propose further adequately designed clinical studies or randomized clinical trials to evaluate the efficacy of TAE in alleviating pain due to plantar fasciitis. Additionally, since imipenem/cilastatin is not approved for use as embolic agents, further investigations are needed.

In conclusion, TAE with imipenem/cilastatin can be effectively employed to treat long-standing refractory cases of plantar fasciitis. The role of TAE can be further confirmed by detailed clinical studies in future.
